# Second-line pembrolizumab versus chemotherapy in Japanese patients with advanced esophageal cancer: subgroup analysis from KEYNOTE-181

**DOI:** 10.1007/s10388-021-00877-3

**Published:** 2021-09-30

**Authors:** Kei Muro, Takashi Kojima, Toshikazu Moriwaki, Ken Kato, Fumio Nagashima, Hisato Kawakami, Ryu Ishihara, Takashi Ogata, Taroh Satoh, Keiichi Iwakami, Shirong Han, Naoyoshi Yatsuzuka, Tomoko Takami, Pooja Bhagia, Toshihiko Doi

**Affiliations:** 1 Department of Clinical Oncology, Aichi Cancer Center Hospital, Nagoya, Japan; 2 Department of Gastrointestinal Oncology, National Cancer Center Hospital East, Kashiwa, Japan; 3 Division of Gastroenterology, University of Tsukuba, Ibaraki, Japan; 4 Department of Head and Neck, Esophageal Medical Oncology, National Cancer Center Hospital, Tokyo, Japan; 5 Department of Medical Oncology, Faculty of Medicine, Kyorin University, Tokyo, Japan; 6 Department of Medicine, Faculty of Medicine, Kindai University, Osaka, Japan; 7 Department of Gastrointestinal Oncology, Osaka International Cancer Institute, Osaka, Japan; 8 Department of Gastrointestinal Surgery, Kanagawa Cancer Center, Yokohama, Japan; 9 Department of Frontier Science for Cancer and Chemotherapy, Osaka University Suita, Osaka, Japan; 10 Department of Medical Oncology, MSD K.K., Tokyo, Japan; 11 Department of Medical Oncology, Merck & Co., Inc., Kenilworth, NJ USA

**Keywords:** Pembrolizumab, Chemotherapy, Esophageal cancer, Cancer immunotherapy, Squamous cell carcinoma

## Abstract

**Background:**

Safe and effective treatments for advanced esophageal cancer are an unmet need in Japan. We report results of a subgroup analysis of Japanese patients enrolled in KEYNOTE-181, a randomized, open-label, phase 3 study of pembrolizumab versus chemotherapy as second-line therapy for patients with advanced or metastatic esophageal cancer whose disease progressed after standard first-line therapy.

**Methods:**

Patients were randomly assigned 1:1 to receive pembrolizumab 200 mg every 3 weeks or investigator’s choice of paclitaxel, docetaxel, or irinotecan. Efficacy was evaluated in all Japanese patients and in those with programmed death ligand 1 combined positive score ≥ 10.

**Results:**

Of the 152 Japanese patients enrolled (pembrolizumab, *n* = 77; chemotherapy, *n* = 75), 150 (98.7%) had squamous cell carcinoma and 79 (52.0%) had combined positive score ≥ 10. At the final analysis, median overall survival was improved among all patients (12.4 vs 8.2 months with pembrolizumab and chemotherapy, respectively; hazard ratio, 0.68; 95% CI 0.48–0.97) and patients with combined positive score ≥ 10 (12.6 vs 8.4 months; hazard ratio, 0.68; 95% CI 0.42–1.10). Fewer patients had any-grade (74.0% vs 95.9%) or grade 3–5 (16.9 vs 50.0%) treatment-related adverse events with pembrolizumab than with chemotherapy.

**Conclusion:**

Consistent with the global trial results, second-line pembrolizumab therapy showed a survival benefit and a favorable safety profile compared with chemotherapy in Japanese patients with advanced esophageal cancer.

**Supplementary Information:**

The online version contains supplementary material available at 10.1007/s10388-021-00877-3.

## Introduction

Esophageal cancer is the ninth most common cancer worldwide [1]. In 2018, 572,000 incident cases of esophageal cancer and 509,000 deaths were reported worldwide [1]; in Japan, more than 22,000 cases and 11,000 deaths were reported [2]. There are two primary histologic subtypes of esophageal cancer, squamous cell carcinoma (SCC) and adenocarcinoma, and the proportion of each varies by region; SCC predominates in Asia and Africa, whereas adenocarcinoma is more prevalent in Europe and North America [3]. In Japan, SCC represents ~ 90% of esophageal cancer cases [4].

Advanced esophageal cancer is associated with a poor prognosis; the 5-year survival rate in Japan is < 30% for those with regional metastases and < 10% for those with distant metastases [2]. The current standard of care for first-line therapy in patients with unresectable advanced or recurrent esophageal cancer in Japan is combination therapy with cisplatin and 5-fluorouracil [5]. For patients whose disease progresses after first-line chemotherapy, treatment options are limited. Taxanes are commonly used as second-line therapy, but responses occur in few patients without a substantial increase in overall survival (OS) [5]. Therefore, Japanese patients with advanced esophageal cancer need more effective treatment options.

Pembrolizumab is a humanized immunoglobulin G4 kappa monoclonal antibody that blocks the interaction between programmed cell death 1 (PD-1) and its ligands, PD-L1 and PD-L2 [6]. Pembrolizumab first demonstrated promising antitumor activity and manageable toxicity in patients with PD-L1-positive advanced esophageal cancer in the phase 1b KEYNOTE-028 study [7]. The objective response rate (ORR) was 30% (*n* = 7 of 23), and nine patients (39%) experienced any-grade treatment-related adverse events (AEs). In the phase 2 KEYNOTE-180 study, pembrolizumab provided durable antitumor activity in patients with heavily pretreated advanced esophageal cancer [8]. Durable responses were also observed in patients with SCC, adenocarcinoma, and high PD-L1 expression (combined positive score [CPS] ≥ 10). The phase 3 KEYNOTE-181 study was conducted to evaluate pembrolizumab versus standard-of-care chemotherapy in patients with previously treated advanced or metastatic esophageal cancer that progressed after first-line therapy [9]. In patients with PD-L1 CPS ≥ 10 tumors, pembrolizumab provided superior OS compared with chemotherapy (hazard ratio [HR], 0.69; 95% confidence interval [CI], 0.52–0.93; *p* = 0.0074). Pembrolizumab was subsequently approved by the US Food and Drug Administration for the treatment of patients with recurrent locally advanced or metastatic SCC of the esophagus whose tumors express CPS ≥ 10 and who experience disease progression after ≥ 1 previous line of systemic therapy [6].

In this analysis, we investigated the antitumor activity of pembrolizumab monotherapy in Japanese patients enrolled in the KEYNOTE-181 study.

## Methods

### Study design

The design of the randomized, open-label, phase 3 KEYNOTE-181 trial has been published [9]. In brief, eligible patients had histologically confirmed SCC or adenocarcinoma of the esophagus, including HER2/neu-negative Siewert type I adenocarcinoma of the esophagogastric junction (EGJ). Patients were randomly assigned 1:1 to receive pembrolizumab 200 mg every 3 weeks or investigator’s choice of standard-of-care chemotherapy with paclitaxel 80–100 mg/m^2^ on days 1, 8, and 15 of each 28-day cycle or docetaxel 75 mg/m^2^ on day 1 of each 21-day cycle; irinotecan is not approved in Japan. Randomization was stratified by histology (SCC vs adenocarcinoma) and geographic region (Asia vs rest of world). The current analysis focuses on the subgroup patients enrolled at Japanese sites.

The study protocol and all amendments were approved by the appropriate ethics committee at each center. The study was conducted in accordance with the protocol, its amendments, and standards of Good Clinical Practice. All patients provided written informed consent.

### Outcomes

Assessment of the primary efficacy and safety outcomes has been described in detail [9]. In the current analysis, efficacy end points were OS, progression-free survival (PFS), ORR, and duration of response (DOR). PFS and tumor response were assessed per RECIST v1.1 by central radiology review. Safety and tolerability, including the incidence of AEs, were evaluated. The severity of AEs was graded according to National Cancer Institute Common Terminology Criteria for Adverse Events version 4.0.

### Statistical analysis

In the Japanese subgroup, efficacy was evaluated in the intention-to-treat population. Efficacy was analyzed in two populations as specified in the protocol: all patients and patients with PD-L1 CPS ≥ 10.

At the time of the protocol-specified final analysis (data cutoff October 15, 2018), two patient deaths had not been included in the data analysis because of an inconsistency in data reporting. A subsequent OS analysis conducted on the October 15, 2018, data cutoff date did include these deaths. The OS results are based on the final analysis and the updated analysis, which includes the deaths of these two patients. In addition, an OS analysis was performed with 4 months of additional follow-up (data cutoff February 13, 2019).

OS and PFS were estimated using the nonparametric Kaplan–Meier method, and treatment differences were assessed using a stratified Cox proportional hazards model with Efron’s method of tie handling. Treatment differences for ORR were assessed using the stratified Miettinen and Nurminen method. The stratification factor for this stratified analysis was tumor histology (SCC vs adenocarcinoma). The data cutoff date for this analysis was February 13, 2019. This trial is registered at ClinicalTrials.gov (NCT02564263).

## Results

### Patients

Of the 628 patients enrolled in KEYNOTE-181, 152 were enrolled in Japan and constituted the Japanese subgroup (pembrolizumab, *n* = 77; chemotherapy, *n* = 75) (Supplementary Table S1). Baseline demographic and disease characteristics were similar between treatment groups (Table 1). Most patients (133; 87.5%) were men, 150 (98.7%) had SCC, and 79 (52.0%) had CPS ≥ 10. Median time from randomization to the data cutoff date was 11.5 months (range 1.0–29.2) in the pembrolizumab group and 8.2 months (range 0.8–32.2) in the chemotherapy group. At the time of data cutoff, all patients had discontinued study treatment, usually because of progressive disease (pembrolizumab, *n* = 66 [85.7%]; chemotherapy, *n* = 58 [78.4%]). Fifty-two (67.5%) patients in the pembrolizumab group received subsequent systemic therapy; paclitaxel was the most common therapy received (*n* = 45 [58.4%]), followed by docetaxel (*n* = 11 [14.3%]) and tegafur/gimeracil/oteracil (*n* = 9 [11.7%]). Thirty-three (44.0%) patients in the chemotherapy group received subsequent systemic therapy; the most common were fluorouracil, tegafur/gimeracil/oteracil, and paclitaxel (*n* = 9 [12.7%] for each); six patients (8%) in the chemotherapy group received subsequent treatment with immune checkpoint inhibitors (three patients received investigational drug [anti-PD-L1 monoclonal antibody TGF-β fusion protein], and one patient each received pembrolizumab, nivolumab, and relatlimab).

**Table 1:** Baseline patient demographics and disease characteristics of the Japanese subgroup

Characteristic	Pembrolizumab *n* = 77	Chemotherapy *n* = 75
Median age, years (range)	67 (50–80)	67 (41–84)
Male, *n* (%)	68 (88.3)	65 (86.7)
ECOG PS, *n* (%)		
0	52 (67.5)	42 (56.0)
1	25 (32.5)	33 (44.0)
Histology, *n* (%)		
SCC	77 (100)	73 (97.3)
AC of esophagus or EGJ Siewert type 1	0	2 (2.7)
PD-L1 combined positive score, *n* (%)^a^		
≥ 10	41 (53.2)	38 (50.7)
< 10	35 (45.5)	37 (49.3)
Disease stage, *n* (%)		
Locally advanced	5 (6.5)	6 (8.0)
Metastatic	72 (93.5)	69 (92.0)
Metastatic staging, *n* (%)		
M0	5 (6.5)	6 (8.0)
M1	72 (93.5)	69 (92.0)
Previous (neo)adjuvant therapy, *n* (%)	7 (9.1)	5 (6.7)
Previous radiation, *n* (%)	45 (58.4)	44 (58.7)
Previous taxane, *n* (%)	21 (27.3)	24 (32.0)

*AC* adenocarcinoma, *CPS* combined positive score, *ECOG PS* Eastern Cooperative Oncology Group performance status, *EGJ* esophagogastric junction, *PD-L1* programmed cell death ligand 1, *SCC* squamous cell carcinoma

^a^One patient was not evaluable because the tumor sample had an inadequate number of cells or no cells

### Overall survival

**Fig. 1:**
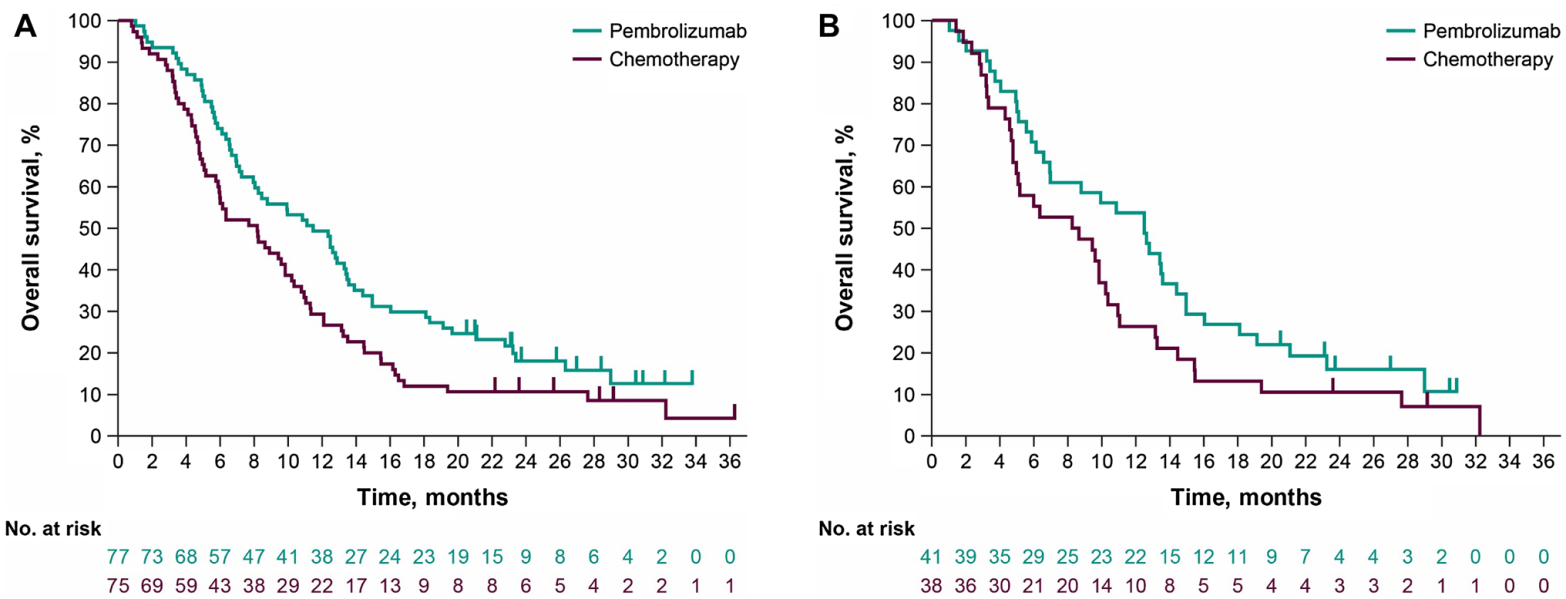
Overall survival in the Japanese subgroup in KEYNOTE-181. **A** All patients and **B** patients with PD-L1 CPS ≥ 10. *CPS* combined positive score, *PD-L1* programmed cell death ligand 1

**Fig. 2:**
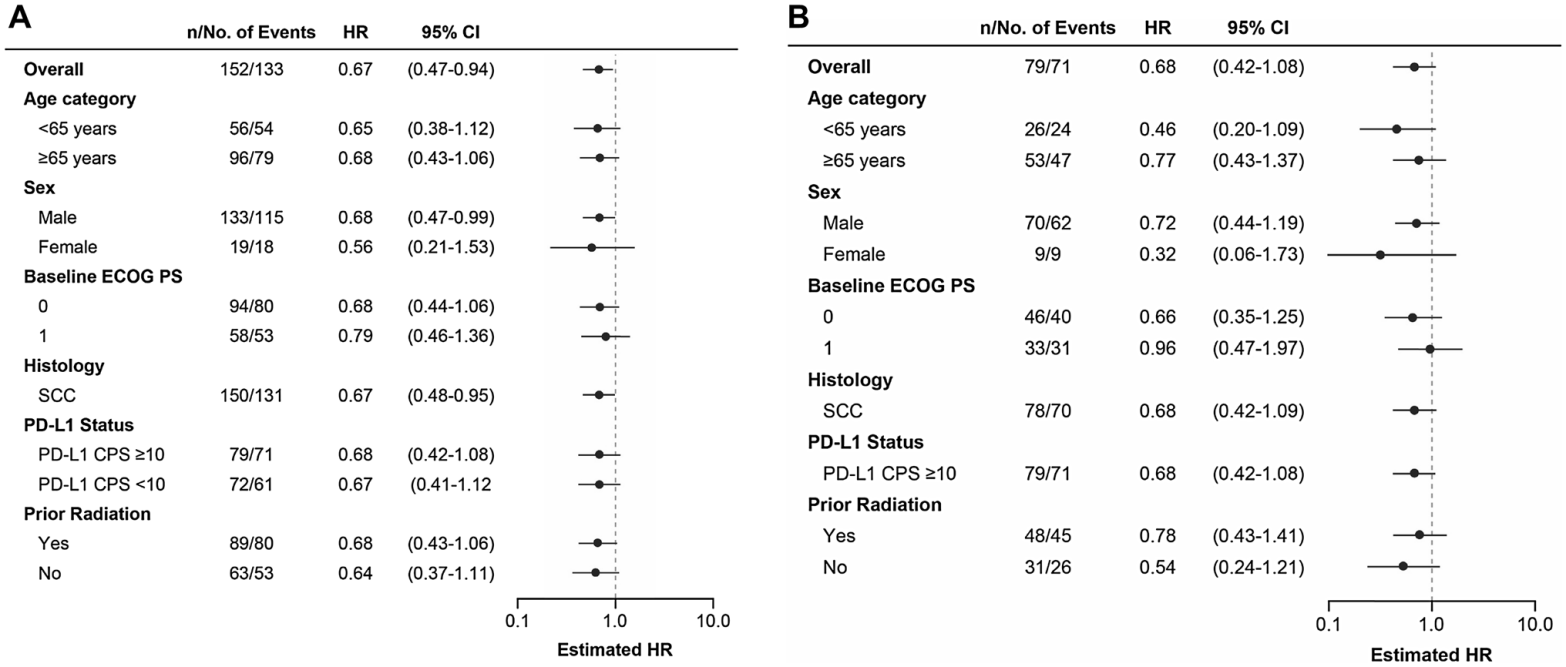
Overall survival by subgroup in Japanese patients in KEYNOTE-181. **A** All patients and **B** patients with PD-L1 CPS ≥ 10. *CI* confidence interval, *CPS* combined positive score, *ECOG PS* Eastern Cooperative Oncology Group performance status, *HR* hazard ratio (pembrolizumab versus chemotherapy), *PD-L1* programmed cell death ligand 1

At the time of the final analysis, 62 patients (80.5%) in the pembrolizumab group and 65 patients (86.7%) in the chemotherapy group had died; median OS was 12.4 and 8.2 months, respectively (HR, 0.68; 95% CI 0.48–0.97) (Supplementary Fig. S1A). The updated analysis showed that 129 patients had died (63 [81.8%] pembrolizumab and 66 [88.0%] chemotherapy). The HR for death was 0.68 (95% CI 0.48–0.96) for pembrolizumab compared with chemotherapy (Supplementary Fig. S2A). With an additional 4 months of follow-up, the HR for death was 0.67 (95% CI 0.47–0.94) (Fig. 1A). Analysis of OS by subgroup factor demonstrated that pembrolizumab was favored over chemotherapy among all subgroups (Fig. 2A).

At the final analysis in the CPS ≥ 10 population, 33 patients (80.5%) in the pembrolizumab group and 33 patients (86.8%) in the chemotherapy group died; median OS was 12.6 and 8.4 months, respectively (HR, 0.68; 95% CI 0.42–1.10) (Supplementary Fig. S1B). The updated analysis showed that 67 patients with CPS ≥ 10 died (34 [82.9%] pembrolizumab and 33 [86.8%] chemotherapy). The HR for death was 0.70 (95% CI 0.43–1.13) for pembrolizumab versus chemotherapy (Supplementary Fig. S2B). With an additional 4 months of follow-up, the HR for death was 0.68 (95% CI 0.42–1.08) (Fig. 1B). Analysis of OS by subgroup factor demonstrated that pembrolizumab was favored over chemotherapy among all subgroups (Fig. 2B).

### Progression-free survival

**Fig. 3:**
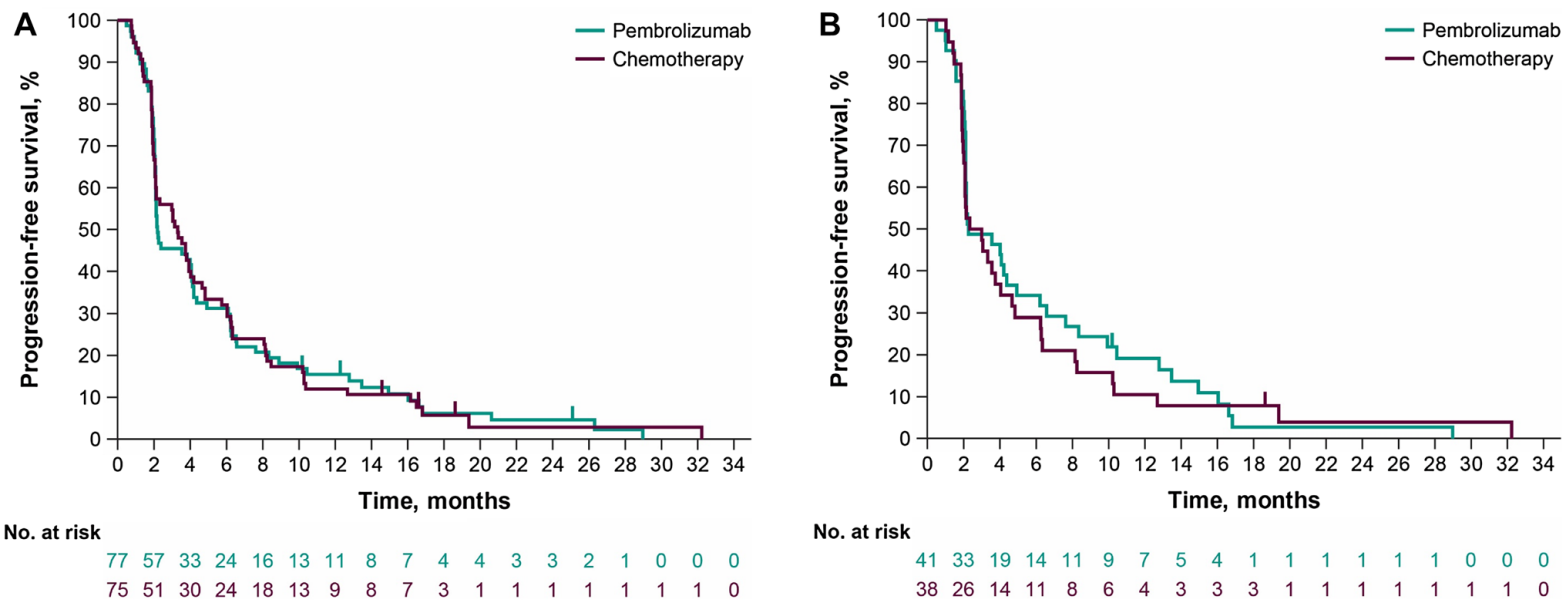
Progression-free survival in the Japanese subgroup in KEYNOTE-181. **A** All patients and **B** patients with PD-L1 CPS ≥ 10. *CPS* combined positive score, *PD-L1* programmed cell death ligand 1

Among all Japanese patients, 74 (96.1%) in the pembrolizumab group and 72 (96.0%) in the chemotherapy group died or experienced disease progression; median PFS was 2.2 and 3.3 months, respectively (HR, 1.00; 95% CI 0.72–1.39) (Fig. 3A). In the CPS ≥ 10 population, 40 patients (97.6%) in the pembrolizumab group and 37 patients (97.4%) in the chemotherapy group died or experienced disease progression; median PFS was 2.3 and 2.7 months, respectively (HR, 0.90; 95% CI 0.57–1.42) (Fig. 3B).

### Tumor response

Among all Japanese patients, 16 of 77 (20.8%) in the pembrolizumab group and 8 of 75 (10.7%) in the chemotherapy group had an objective response (Table 2). The median DOR was 8.4 months (range 2.1+ to 14.8 months) in the pembrolizumab group and 10.7 months (4.1+ to 16.8+ months) in the chemotherapy group. In the CPS ≥ 10 population, 11 of 41 patients (26.8%) in the pembrolizumab group and 3 of 38 patients (7.9%) in the chemotherapy group had an objective response; median DOR was 8.4 months (range 2.1+ to 14.8 months) and 10.7 months (4.3 to 16.8+ months), respectively.

**Table 2:** Antitumor activity in Japanese patients by subgroup

Best overall response	All patients		PD-L1 CPS ≥ 10	
	Pembrolizumab *n* = 77	Chemotherapy *n* = 75	Pembrolizumab *n* = 41	Chemotherapy *n* = 38
ORR (CR + PR)	16 (20.8)	8 (10.7)	11 (26.8)	3 (7.9)
CR	3 (3.9)	0	2 (4.9)	0
PR	13 (16.9)	8 (10.7)	9 (22.0)	3 (7.9)
SD	19 (24.7)	32 (42.7)	9 (22.0)	15 (39.5)
Disease control rate (CR + PR + SD)	35 (45.5)	40 (53.3)	20 (48.8)	18 (47.4)
PD	40 (51.9)	27 (36.0)	19 (46.3)	16 (42.1)
No assessment/nonevaluable^a^	2 (2.6)	8 (10.7)	2 (4.9)	4 (10.5)

*CPS* combined positive score, *CR* complete response, *ORR* objective response rate, *PR* partial response, *SCC* squamous cell carcinoma, *SD* stable disease

^a^Captures patients for whom no postbaseline assessments were performed because of death, withdrawal of consent, loss to follow-up, or start of new anticancer therapy and patients who had ≥ 1 postbaseline tumor assessment, none of which was evaluable for response determination (e.g., not all target lesions captured)

### Adverse events

Most patients experienced ≥ 1 AE: 71 patients (92.2%) in the pembrolizumab group and 73 patients (98.6%) in the chemotherapy group (Table 3). Treatment-related AEs were reported in 57 patients (74.0%) in the pembrolizumab group and 71 patients (95.9%) in the chemotherapy group; grade 3–5 events were reported in 13 patients (16.9%) and 37 patients (50.0%), respectively. Two patients died of treatment-related AEs, one in each treatment group. Immune-mediated AEs were reported in 24 patients (31.2%) in the pembrolizumab group and four patients (5.4%) in the chemotherapy group (Supplementary Table S2). The most common immune-mediated AEs (≥ 5%) with pembrolizumab were hypothyroidism (*n* = 9; 11.7%) and pneumonitis (*n* = 6; 7.8%).

**Table 3:** Adverse events in the Japanese subgroup

Event, *n* (%)	Pembrolizumab *n* = 77	Chemotherapy *n* = 74
≥ 1 AE	71 (92.2)	73 (98.6)
Grade 3–5	29 (37.7)	44 (59.5)
Led to discontinuation	7 (9.1)	9 (12.2)
Serious	19 (24.7)	27 (36.5)
Serious and led to discontinuation	5 (6.5)	6 (8.1)
Led to death	3 (3.9)	3 (4.1)
≥ 1 treatment-related AE	57 (74.0)	71 (95.9)
Grade 3–5	13 (16.9)	37 (50.0)
Led to discontinuation	7 (9.1)	6 (8.1)
Serious	14 (18.2)	15 (20.3)
Serious and led to discontinuation	5 (6.5)	3 (4.1)
Led to death^a^	1 (1.3)	1 (1.4)

*AE* adverse event

^a^One patient in the pembrolizumab group died of treatment-related pneumonitis and one patient in the chemotherapy group died of treatment-related aspiration pneumonitis

## Discussion

Prognosis is poor among Japanese patients with advanced esophageal cancer, and treatment options are limited after disease progression on first-line chemotherapy. In the current subgroup analysis of the phase 3 KEYNOTE-181 study, second-line therapy with pembrolizumab prolonged OS among Japanese patients with esophageal cancer compared with chemotherapy and demonstrated a robust response rate. The safety and tolerability profile favored pembrolizumab, with substantially more treatment-related AEs occurring among chemotherapy-treated patients. Further, the results in Japanese patients were consistent with the data reported in the global population of KEYNOTE-181, though there were several differences in baseline characteristics between the populations; most Japanese patients had Eastern Cooperative Oncology Group performance status 0 (61.8%), SCC (98.7%), and PD-L1 CPS ≥ 10 (52.0%) compared with the global population (38.5, 63.9, and 35.4%, respectively) [9].

Second-line therapy with chemotherapy, primarily taxanes, is the current treatment standard in Japan for patients with advanced esophageal cancer; however, the survival benefit is limited because of high toxicity [5]. Treatment with single-agent docetaxel and paclitaxel offered median OS of 8.1 months and 10.4 months, respectively, and a high incidence of neutropenia (87.8 and 79.2%, respectively) [10, 11]. Trials of combination chemotherapy have studied docetaxel plus capecitabine or gemcitabine, docetaxel plus nedaplatin, and docetaxel with cisplatin plus fluorouracil. Median OS ranged from 5.9 to 11.1 months, with an AE profile similar to that of single-agent chemotherapy trials [12–15]. Given that similar results have been reported in patients with esophageal cancer and patients with gastric/gastroesophageal junction (GEJ) cancer [16], it is reasonable to consider data from large phase 3 studies of second-line chemotherapy for gastric/GEJ cancer. These trials have found median OS to be shorter than 6–7 months [16–18], which is slightly shorter than we observed in chemotherapy-treated patients in the current analysis (8 months). Trials with targeted therapies in esophageal cancer, including gefitinib monotherapy, have failed, which is why chemotherapy remains the standard of care [19]. Therefore, KEYNOTE-181 is the only large phase 3 randomized trial in this population of patients with esophageal cancer who have high unmet need.

PD-1 or PD-L1 inhibition has the potential to provide clinically meaningful improvement in survival and to maintain or improve quality of life outcomes among Japanese patients with esophageal cancer. Takahashi et al. [20] analyzed data from Japanese patients enrolled in ATTRACTION-3, which was a global, randomized, open-label, phase 3 study that evaluated the efficacy and safety of nivolumab versus chemotherapy as second-line treatment in patients with advanced ESCC who were refractory to or intolerant of standard chemotherapy, regardless of PD-L1 expression. OS in the Japanese population tended to be longer in the nivolumab group than in the chemotherapy group (median, 13.4 vs 9.4 months; HR, 0.77; 95% CI 0.59–1.01); the ORR in the Japanese population was 22.4%. In the current analysis from KEYNOTE-181 in Japanese patients, pembrolizumab demonstrated a positive trend for survival benefit in all patients (all but two patients had SCC) and for patients with CPS ≥ 10, with HRs for OS ranging from 0.67 to 0.70. Further, OS was consistent regardless of PD-L1 status (Fig. 2A). Of note, this trend is different from that reported in the global population [9]; more analyses on medical background, baseline characteristics, and subsequent therapy are needed to elucidate this observation. In addition, response rates in Japanese patients treated with pembrolizumab nearly doubled in all patients and tripled in the CPS ≥ 10 population compared with chemotherapy, supporting the use of pembrolizumab in Japanese patients with advanced esophageal cancer. Overall, data with PD-1/PD-L1 inhibitors in Japanese patients with advanced esophageal cancer are limited.

In the KEYNOTE-181 study, patients were eligible for enrollment if they had previously received first-line therapy with a taxane, such as carboplatin plus paclitaxel or docetaxel plus fluorouracil plus cisplatin. Kaplan–Meier estimates for OS in both groups of Japanese patients with SCC who did not previously receive taxane therapy were consistent with those reported for the entire Japanese SCC population, regardless of previous taxane therapy (data not shown). No remarkable differences in efficacy in the Japanese population were observed in the chemotherapy group based on previous taxane therapy, but patients who previously received taxane therapy did achieve a relatively lower ORR (1/24; 4.2%) than those who did not (7/49; 14.3%) (data not shown). Moreover, it is possible that the discrepancy between OS and PFS occurred because of the relatively higher rate of patients in the pembrolizumab group who received subsequent therapy (SCC: 52/77 patients in the pembrolizumab group vs 31/71 in the chemotherapy group; CPS ≥ 10: 27/41 patients in the pembrolizumab group vs 18/38 in the chemotherapy group).

The safety profile of pembrolizumab was consistent between Japanese patients and the global population [9]. Treatment-related AEs were reported in 74.0% of patients in the Japanese population and 64.3% of patients in the global population; grade 3–5 treatment-related AEs were reported in 16.9 and 18.2%, respectively; treatment-related AEs that led to discontinuation were reported in 9.1 and 6.1%, respectively; and treatment-related AEs that led to death were reported in 1.3 and 1.6%, respectively [9].

The primary limitation of the present report is that we present a subgroup analysis of a larger clinical trial, though Japanese patients constituted approximately one-fourth of the global KEYNOTE-181 study.

Second-line pembrolizumab therapy improved OS compared with chemotherapy in the Japanese subgroup of patients with previously treated advanced or metastatic esophageal cancer, which was consistent with findings in the global population. A positive OS trend was observed in those with PD-L1 CPS ≥ 10. OS and PFS in Japanese patients were comparable with OS and PFS in the global population. Pembrolizumab also demonstrated a favorable safety profile compared with chemotherapy in the Japanese subgroup. These data suggest that pembrolizumab warrants consideration as a second-line treatment option for Japanese patients with unresectable recurrent or advanced esophageal cancer.

## Supplementary Information

Below is the link to the electronic supplementary material.

Supplementary file1 (PDF 760 KB)

## Data Availability

Merck Sharp & Dohme Corp., a subsidiary of Merck & Co., Inc., Kenilworth, NJ, USA (MSD) is committed to providing qualified scientific researchers access to anonymized data and clinical study reports from the company’s clinical trials for the purpose of conducting legitimate scientific research. MSD is also obligated to protect the rights and privacy of trial participants, and as such, has a procedure in place for evaluating and fulfilling requests for sharing company clinical trial data with qualified external scientific researchers. The MSD data-sharing website (available at: http://engagezone.msd.com/ds_documentation.php) outlines the process and requirements for submitting a data request. Applications will be promptly assessed for completeness and policy compliance. Feasible requests will be reviewed by a committee of MSD subject matter experts to assess the scientific validity of the request and the qualifications of the requestors. In line with data privacy legislation, submitters of approved requests must enter into a standard data-sharing agreement with MSD before data access is granted. Data will be made available for request after product approval in the US and EU or after product development is discontinued. There are circumstances that may prevent MSD from sharing requested data, including country or region-specific regulations. If the request is declined, it will be communicated to the investigator. Access to genetic or exploratory biomarker data requires a detailed, hypothesis-driven statistical analysis plan that is collaboratively developed by the requestor and MSD subject matter experts; after approval of the statistical analysis plan and execution of a data-sharing agreement, MSD will either perform the proposed analyses and share the results with the requestor or will construct biomarker covariates and add them to a file with clinical data that is uploaded to an analysis portal so that the requestor can perform the proposed analyses.

## Abbreviations

AE: Adverse event
CI: Confidence interval
CPS: Combined positive score
CR: Complete response
DOR: Duration of response
EGJ: Esophagogastric junction
GEJ: Gastroesophageal junction
HR: Hazard ratio
ORR: Objective response rate
OS: Overall survival
PD-1: Programmed cell death 1
PD-L1: Programmed death ligand 1
PD-L2: Programmed death ligand 2
PFS: Progression-free survival
PR: Partial response
SCC: Squamous cell carcinoma
SD: Stable disease
TRAE: Treatment-related adverse event
