# Supplementary material for: Second-line pembrolizumab versus chemotherapy in Japanese patients with advanced esophageal cancer: subgroup analysis from KEYNOTE-181
Source: Esophagus. 2021 Sep 30;19(1):137–45. doi: 10.1007/s10388-021-00877-3 (PMC8739314; doi:10.1007/s10388-021-00877-3)
Supplement: Supplementary file 1 — Supplementary file1 (PDF 760 KB) [file 10388_2021_877_MOESM1_ESM.pdf]

Online Resource 1 for *Esophagus*

Second-line pembrolizumab versus chemotherapy in Japanese patients with advanced esophageal cancer: subgroup analysis from KEYNOTE-181

Kei Muro, Takashi Kojima, Toshikazu Moriwaki, Ken Kato, Fumio Nagashima, Hisato Kawakami, Ryu Ishihara, Takashi Ogata, Taroh Satoh, Keiichi Iwakami, Shirong Han, Naoyoshi Yatsuzuka, Tomoko Takami, Pooja Bhagia, Toshihiko Doi

Corresponding author:

Kei Muro, MD

Department of Clinical Oncology

Aichi Cancer Center Hospital

Email: kmuro@aichi-cc.jp

Supplementary Table S1 Patient disposition of Japanese subgroup

n (%)	Pembrolizumab<br>*n* = 77	Chemotherapy<br>*n* = 75
Started	77	74
Discontinued	77 (100)	74 (100)
Adverse events	7 (9.1)	9 (12.2)
Clinical progression	3 (3.9)	6 (8.1)
Patient decision	0	1 (1.4)
Physician decision	1 (1.3)	0
Progressive disease	66 (85.7)	58 (78.4)

Supplementary Table S2 Immune-mediated adverse events and infusion-related reactions in Japanese subgroup

Event, *n* (%)	Pembrolizumab *n* = 77	Chemotherapy *n* = 74
Immune-mediated adverse events	24 (31.2)	4 (5.4)
Hypothyroidism	9 (11.7)	1 (1.4)
Pneumonitis	6 (7.8)	1 (1.4)
Infusion-related reaction	3 (3.9)	0
Colitis	2 (2.6)	0
Interstitial lung disease	2 (2.6)	1 (1.4)
Erythema multiforme	1 (1.3)	0
Guillain-Barré syndrome	1 (1.3)	0
Hyperthyroidism	1 (1.3)	0
Polymyositis	1 (1.3)	0
Rash	1 (1.3)	0
Type 1 diabetes mellitus	1 (1.3)	0
Dermatitis bullous	0	1 (1.4)

Supplementary Fig S1 Overall survival at the final analysis in the Japanese subgroup. (**A**) All patients and (**B**) patients with PD-L1 CPS ≥10. The data cutoff date for this analysis was October 15, 2018. *CPS* combined positive score, *PD-L1* programmed cell death ligand 1.

A

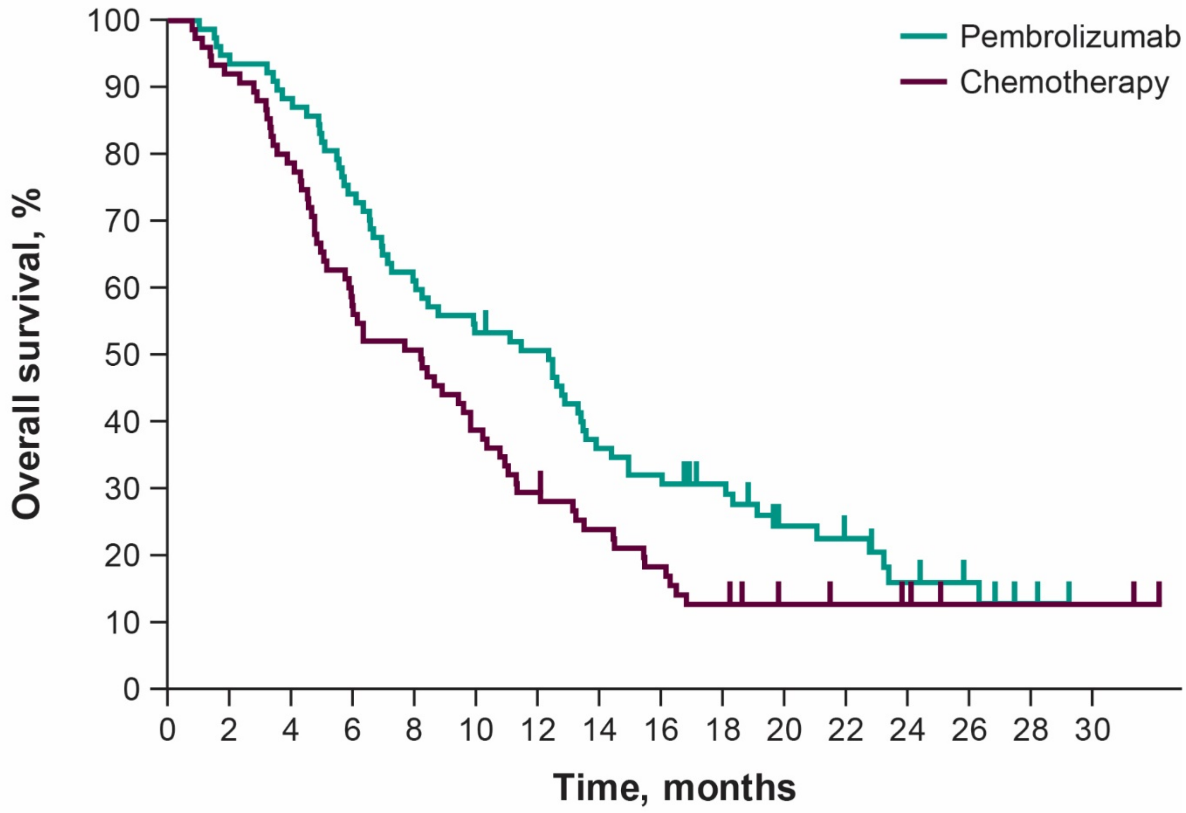

No. at risk

77	73	68	57	47	41	38	27	24	20	13	11	7	5	2	0
75	69	59	43	38	29	22	17	13	9	6	5	4	2	2	2

B

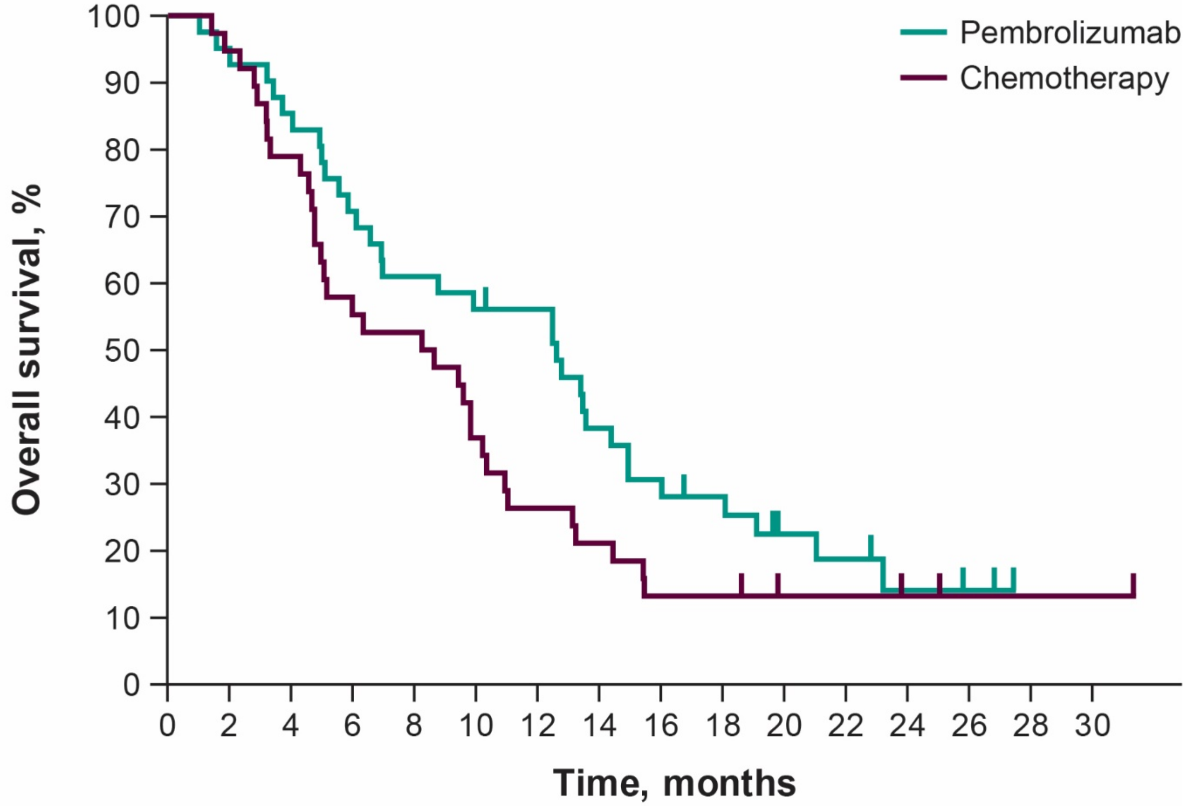

No. at risk

41	39	35	29	25	23	22	15	12	10	6	5	3	2	0	0
38	36	30	21	20	14	10	8	5	5	3	3	2	1	1	1

Supplementary Fig S2 Overall survival at updated analysis in the Japanese subgroup. (A) All patients and (B) patients with PD-L1 CPS ≥10. The data cutoff date for this analysis was October 15, 2018. *CPS* combined positive score, *PD-L1* programmed cell death ligand 1.

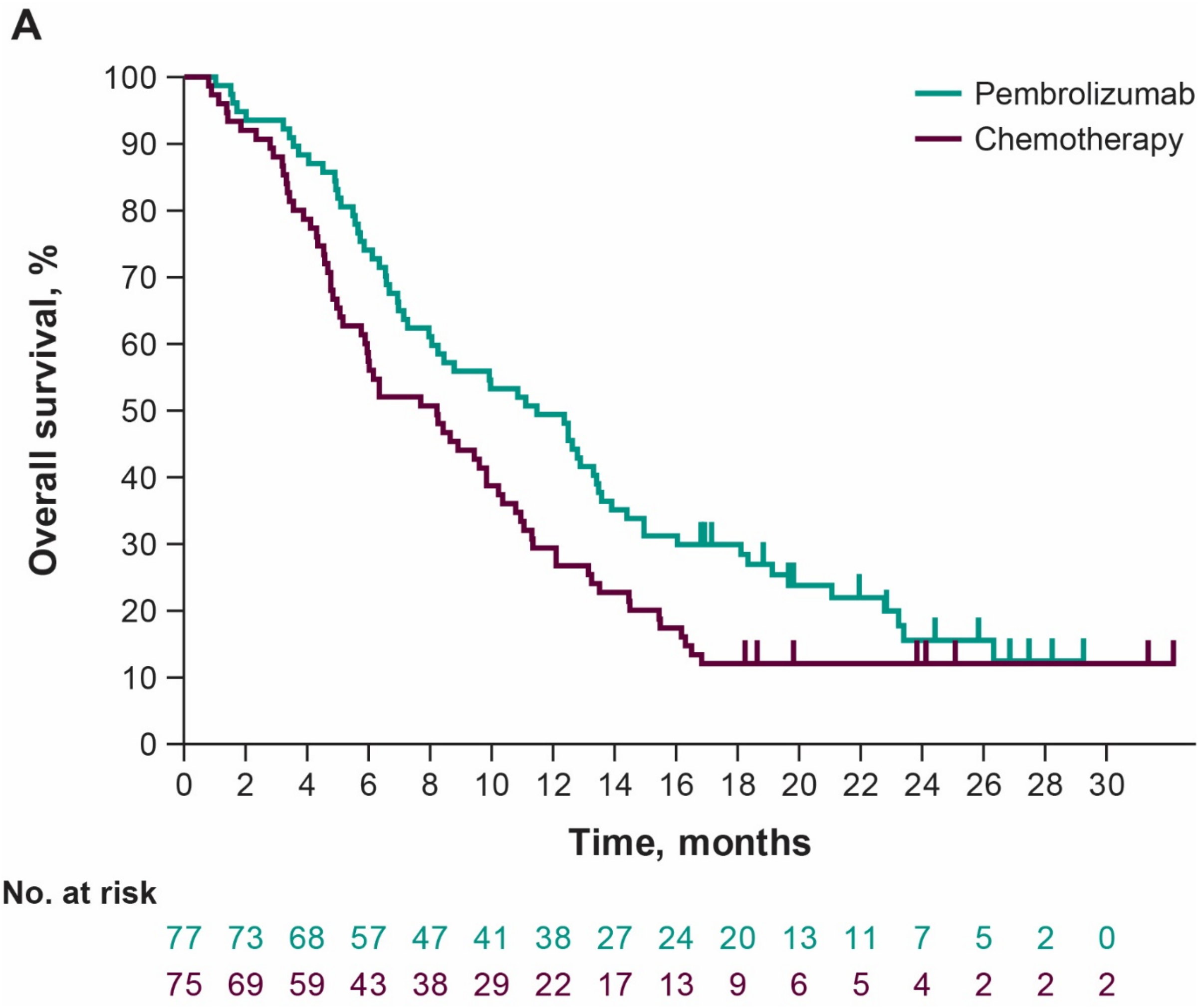

B

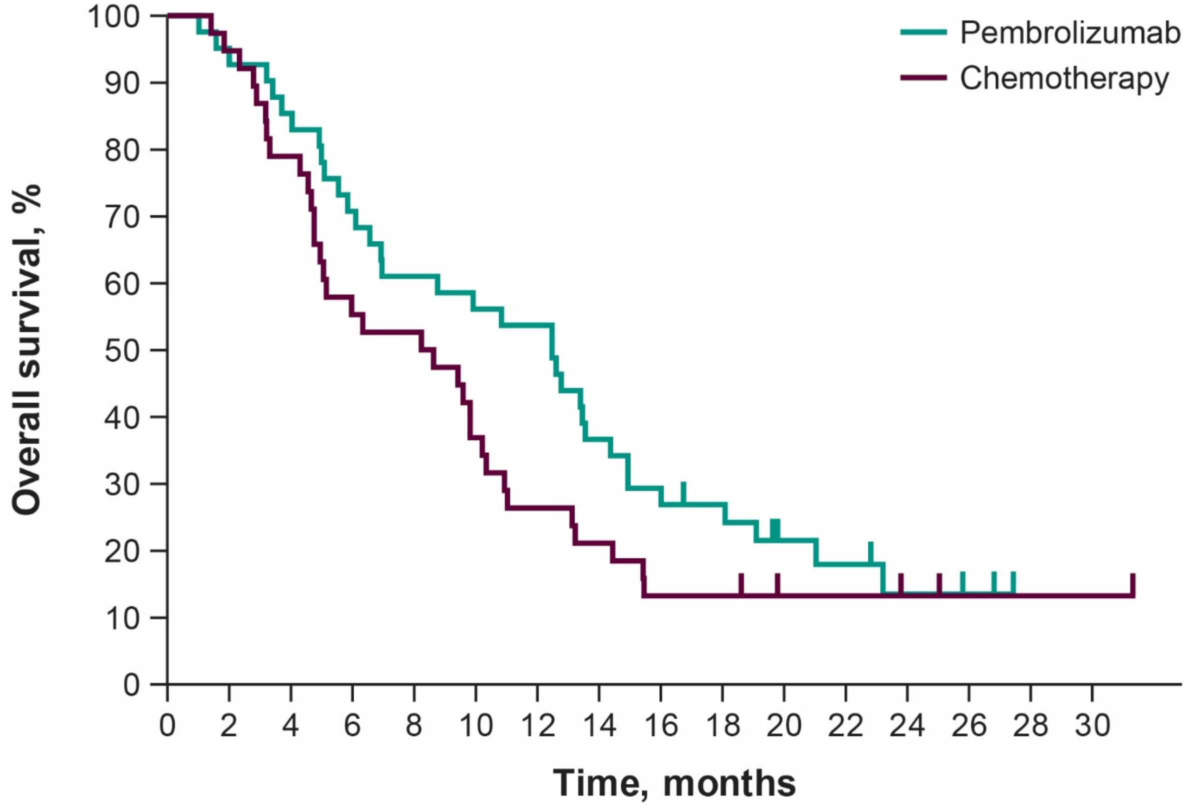

No. at risk

41	39	35	29	25	23	22	15	12	10	6	5	3	2	0	0
38	36	30	21	20	14	10	8	5	5	3	3	2	1	1	1
